# Biodiversity, Ecology and Distribution of Mediterranean Charophytes in Southern Italy

**DOI:** 10.3390/plants12193434

**Published:** 2023-09-29

**Authors:** Alessandro Bellino, Daniela Baldantoni

**Affiliations:** Department of Chemistry and Biology “Adolfo Zambelli”, University of Salerno, Via Giovanni Paolo II, 132, 84084 Fisciano, Salerno, Italy

**Keywords:** characeae occurrence, charophyte conservation, *Chara* genus, ecological niche, Mediterranean area

## Abstract

Charophytes are amongst the most endangered primary producers in freshwater and coastal ecosystems. In spite of the extensive research on the group and its ecological and conservational relevance, scarce information is available on Mediterranean environments, especially rivers and small water reservoirs, where charophytes face challenging summer droughts and changes in hydrological regimes, as well as pervasive anthropogenic pressures. This research aimed, through repeated field observations, detailed analyses of population traits, and extensive characterization of the colonized environments, to foster an understanding of the distribution, biodiversity, and ecology of charophytes in an area of exceptional environmental value and that is still uninvestigated in relation to its charophyte flora, the southern Campania region (Italy). Overall, 17 populations were discovered, belonging to 4 taxa of the *Chara* genus: *C. globularis*, *C. gymnophylla*, *C. vulgaris*, and *C. vulgaris* var. *papillata*, reduced to 12 populations and to the first 3 taxa by the end of the study. The species occupied different ecological niches and colonized environments such as rivers and small ponds, with environment-dependent morphotypes. The occurrence of few taxa with a wide distribution, often forming ephemeral populations, suggests ongoing constraints on charophyte biodiversity in the area, favoring opportunistic species that are able to benefit from temporary refugia.

## 1. Introduction

Characeae (Charophyceae, Charales), hereafter referred to as charophytes, are a worldwide distributed group of macrophytes that comprise several species colonizing different Mediterranean aquatic habitats [1]. They derived their common name of “stoneworts” [2] from the stony external texture that several species acquire as a result of encrustation of their surface, mainly with calcium carbonate [3], and they are included in a number of red lists [4,5,6], since they represent the most endangered group of macrophytes [3,7,8] and one of the most threatened groups of organisms in Europe [1,9,10]. Many of the habitats dominated by charophytes are also of community interest [7] according to the Habitats Directive 92/43/EEC [11], and their conservation is thus considered under the Water Framework Directive 2000/60/EC [12]. For these reasons, intensive monitoring, protection, and restoration actions, both indirectly through improving the habitat conditions and directly through transplantations, currently represent priorities in supporting endangered species of charophytes [13].

The biology of these submerged macroalgae, characterized by a green thallus with a complex morphology [14], is still the subject of intense research, especially in relation to their molecular phylogeny and taxonomy [15]. Charophytes appeared nearly 460 million years ago [3] and are now represented by 476 species [5,16] assigned, based on morphological criteria, to three cosmopolitan genera (*Chara*, *Nitella*, *Tolypella*) and three distribution-limited genera (*Lamprothamnium*, *Nitellopsis*, *Lychnothamnus*) in the Characeae family. According to molecular analyses, their taxonomic classification is currently being extensively revised [17].

Charophytes are widely distributed in freshwater as well as in brackish waters, from tropical to polar regions [3,4,18,19]. They represent a common component of the benthic macrophytic community in the littoral zone of oligo- and mesotrophic water bodies worldwide [17,20,21], and several taxa are considered indicative of a high ecological status of the colonized environments [5,22,23,24]. Lakes, rivers, and permanent streams are suitable for the maintenance of permanent populations of charophytes, in some cases represented by perennial species, whereas intermittent pools, ponds, ditches, and lake edges, characterized by marked fluctuations in water level offer suitable habitats for species capable of withstanding periods of desiccation [20]. In spite of the large ecological plasticity of charophytes [20], they have a high sensitivity to physical and chemical environmental stresses [15,25], mainly water pollution [7], mostly associated with agricultural run-off and urbanization [3]. In this context, eutrophication is probably the most important threat causing the decline of several species [3,9,26], since only a few species are able to tolerate eutrophic waters [17]. As a consequence, a progressive decline in the abundance, occurrence, and diversity of charophytes over the past decades, due to anthropogenic impacts, has been observed worldwide [27,28].

In freshwater ecosystems, charophytes are responsible for a high primary production, providing trophic and spatial niches for other aquatic organisms [3,29,30,31]. Moreover, by counteracting resuspension of sediment particles [3,29,32], by acting as nutrient sinks [21,29,33], and by hampering phytoplankton biomass growth [4,10], charophytes participate in the establishment and maintenance of clear-water states [3,29]. As a consequence, charophytes characterize the pattern of ecological successions (in which they often represent pioneer species [6]) and the community structure of several types of water bodies [3,29]. Their conservation and the promotion of the establishment of charophyte meadows can thus prove pivotal in ensuring freshwater ecosystem functioning and dynamics [18,21].

In spite of their crucial role in aquatic ecosystems and their strong positive impacts on their environment [10,29,30,34], information on stonewort occurrence, distribution, and ecological requirements is still insufficient, especially in the Mediterranean area [1,35]. In fact, in this unique biodiversity hot spot, efforts to study the diversity and distribution of stoneworts have been limited to small geographical areas and focused mainly on freshwater lakes [1,7,36] and wetlands [1,37], with recent attempts at investigating the role of man-made habitats such as farm ponds [23] in creating refugia for these species. Remarkably, the typical habitat dominated by charophytes in inland waters, included in the Habitats Directive 92/43/EEC [11] with code 3140—Hard oligo-mesotrophic waters with benthic vegetation of *Chara* spp., is only occasionally reported [22], with large areas such as the entire Campania region ( 13,671 km^2^) having a single known station (Natura 2000 site code IT8040007). In this context any information, e.g., the description of novel populations or the revision of species distributions, is valuable for shedding light on the ecology and biogeography of charophytes [35], especially in areas where even historical records are scarce, such as most of southern Italy.

Since environmental factors (particularly water physico-chemical characteristics [28]) have a considerable impact on both the distribution and morphological traits of charophytes [3,4,34,38], this study aimed to improve our knowledge about the occurrence, distribution, and ecology of charophytes in the Mediterranean area, focusing on the “Cilento, Vallo di Diano e Alburni” National Park (Province of Salerno, southern Italy). In particular, the morphological, biochemical, and ecological characteristics of each population were investigated and related to the geographical, hydrogeological, and geochemical characteristics of their finding sites, being used to define the ecological niche of each species. The relationships among charophytes occurrence, distribution, and ecology not only contribute to establishing how species traits are shaped by environmental characteristics [38], pivotal in a conservation context [7], but are also useful in modeling [9] the temporal changes in the distribution of these priority species [3]. In turn, a better understanding of these topics has relevance for the conservation and restoration of aquatic ecosystems. Indeed, to satisfy the implementations of the Global Strategy for Plant Conservation, the Habitats Directive 92/43/EEC [11], and the Water Framework Directive 2000/60/EC [12], it may be necessary to develop ecology-based strategies to manage water bodies where charophytes occur [7,18].

## 2. Materials and Methods

### 2.1. Study Design

Several field trips were performed in the years 2015–2018 to verify charophyte occurrence and distribution in different freshwater habitats (rivers, lakes, brooks, ponds, pools, springs, ditches, swamps, artificial water reservoirs…) of the “Cilento, Vallo di Diano e Alburni” National Park. The park, located in the southernmost part of the Campania region, in Italy, in the heart of the Mediterranean Basin, has an area of ∼180,000 ha and was founded in 1991 for its unique flora and fauna biodiversity [39,40]. In 1998, it was included in the UNESCO World Heritage List for its cultural landscape of outstanding value and in 2010 it was recognized as geopark of the European and Global Geopark Network due to its geological heritage, characterized by a high degree of diversity [39,40].

Each charophyte population found was georeferenced and sampled during the warm season, in May 2018, in order to find fertile specimens for easier identification. From the green portion of fully developed specimens from each population, the stipulode types and cortification traits of internodes and branchlet segments were investigated, while measurements of the length and diameter of internodes, spine cells, corticated and ecorticated branchlet segments, oosporangia (=fertilized oogonia, *sensu* Soulié-Märsche and García [41]), coronula, and antheridia were performed (Section 2.2). Only fully opaque oosporangia developing on the branchlets of at least the fifth internode from the apex were selected for measurements, in order to ensure a comparable developmental stage [42]. The abbreviations adopted for the morphological parameters are reported in Table 1. With the same samples, measurements of carbonate encrustation, pigment concentrations and epiphyte diatom mass were carried out (Section 2.2).

The ecological niche occupied by each population was characterized in terms of the topographical properties and physico-chemical characteristics of both water and sediments. In particular, information on geographical coordinates, elevation, and exposure were acquired using a handheld GPS receiver (GPSMAP 62s, Garmin, Olathe, KS, USA) with a horizontal resolution of 1–3 m. Moreover, measurements of water electrical conductivity, pH, dissolved oxygen, phytoplankton pigment profile, as well as total organic carbon, inorganic carbon, total nitrogen, potentially toxic elements, and anion concentrations were performed (Section 2.3). In sediments, measurements of pH and organic matter concentration, as well as analyses of the mineralogical characteristics and particle distribution size, were carried out (Section 2.4). The rationale for analyzing the concentrations of potentially toxic elements in water and not in sediments was based on the element uptake in charophytes mostly occurring through direct transport from water [28], either through the outer cells of the branchlets and stems [3] or through the fine rhizoids accessing interstitial water [43,44].

### 2.2. Algae Identification and Analysis

Since the charophyte populations were always found in water no deeper than chest waders (1.5 m), samples were collected by hand from the external and central part of the algae meadow [19]. Samples from each population were transported to the laboratory in plastic buckets with their own water and processed for further analyses within one day from collection. Morphological characteristics were employed as diagnostic features for stonewort identification at the species level [19,38], the classification of which relied on the dichotomous keys of Bazzichelli and Abdelahad [45] and Urbaniak and Gąbka [46], and on the current nomenclature reported in AlgaeBase [16] and in the World Checklist of Selected Plant Families [47]. Morphological analyses were performed through image analysis, using ImageJ 1.8.0 software, on 5 thalli per population, fixed and preserved in formalin-acetic acid-alcohol (FAA) solution at 4 °C [48] in line with similar studies [4,14]. The images were taken either using a Df (Nikon Imaging, Tokio, Japan) camera equipped with a 58 mm f/1.4 (Voigtländer, Fürth, Germany) lens, for the measurement of internode length on graph paper, or a CoolSnap K4 (Photometrics, Tucson, AZ, USA) camera mounted on a Dialux 20 (Leitz, Oberkochen, Germany) microscope, for all the other parameters. Microscope images were taken at 25×, 100×, 250×, and 400× magnifications.

Gravimetric analyses were employed for measuring carbonate encrustation, according to Sviben et al. [49]. Specifically, 3–11 thalli (approximately 5 g f.w.) per population were dried at 75 °C in an incubator (ISCO 9000, Sil.Mar Instruments, Milan, Italy) until constant weight; the dried samples were weighed and treated with 16% HCl solution for 15 min, in order to remove ${\mathrm{CaCO}}_{3}$ incrustations, then washed several times with distilled water, re-dried at 75 °C and weighed again. The mass of carbonates precipitated on the surface of stoneworts was thus calculated as the difference between the initial and the final weights.

The extraction of photosynthetic pigments was performed on 5 thalli per population, pulverized under liquid nitrogen, by 100% acetone at −18 °C for 1 day [50]. Chlorophylls (chlorophyll *a* and chlorophyll *b*), pheophytins (pheophytin *a* and pheophytin *b*) and total carotenoids were quantified through UV-Vis spectrophotometry and spectra deconvolution [50] using the Gauss Peak Spectra fitting [51] technique. In particular, the absorbance spectra of the centrifuged (5000 rpm for 10 min) samples were recorded in the range 350–750 nm using a UV-Vis 1800 (Shimadzu, Kyoto, Japan) spectrometer and were deconvoluted using the set of equations for Chlorophyta provided by Küpper et al. [51]. Micro-molar concentrations were then referred to the fresh weights of thalli.

For epiphyte diatom mass analysis [49], weighed samples of 5 thalli per population, fixed in FAA, were placed in 50 mL polyethylene centrifuge tubes and sonicated in a Labsonic LBS1-3 (FALC, Bergamo, Italy) ultrasonic cleaner, in FAA solution, for 30 min. Thalli were then removed, the FAA solution was centrifuged at 4000 rpm for 20 min, and the supernatant discarded. A drop of 16% HCl was added to the samples (to remove carbonates), which were then treated with 2 mL of 30% $H_{2}O_{2}$ solution for 7 days to remove organic matter and, after a second centrifugation and removal of the supernatant, were dried at 75 °C in an incubator (ISCO 9000, Sil.Mar Instruments, Italy). Epiphyte diatom biomass was then estimated through weighing of the diatom frustules, referring this to each thallus fresh weight. Although this technique may underestimate the diatom biomass due to difficulties in detaching frustules from charophyte thalli [52], the identical conditions adopted for all the specimens ensured the comparability of results among charophyte populations.

### 2.3. Water Analysis

Water sampling and measurements were performed in triplicate across the algae meadow. The water electrical conductivity (EC) as well as the water pH and dissolved oxygen ($O_{2}$) were measured in situ using portable field measurement equipment (HI9835 and HI9147, respectively, Hanna Instruments, Italy). In addition, water samples were collected for the analysis of phytoplankton pigment profile (3 × 1.5 L), total organic carbon (TOC), inorganic carbon (IC) and total nitrogen (TN) concentrations (3 × 50 mL), potentially toxic element (PTE) concentrations (3 × 50 mL, acidified to pH = 2 in the field with 65% ${\mathrm{HNO}}_{3}$), and anion concentrations (3 × 15 mL). Samples were kept in cold and dark conditions until the extraction of photosynthetic pigments, carried out on the same day back in the laboratory or were frozen at −18 °C for all other analyses.

Water samples for phytoplankton pigment profiling were vacuum-filtered on glass filters (Ø 47 mm, 1.2 μm pore size; Whatman, Little Chalfont, UK), from which pigments were extracted with 100% acetone at −18 °C until the analysis, carried out 1 week later. Pigment profile as well as chlorophyll (chlorophyll *a* and chlorophyll *b*), pheophytin (pheophytin *a* and pheophytin *b*), and total carotenoid concentrations were measured on the same extracts, as described in the Section 2.2.

TOC, IC, and TN analyses were carried out using a V-CNS TOC analyzer (Shimadzu, Japan), measuring TOC as the difference between total C and IC.

Total PTE concentrations were determined by means of inductively coupled plasma optical emission spectrometry (Optima 7000DV, PerkinElmer, Waltham, MA, USA), using a PTFE Gem-Cone nebulizer and Cyclonic chamber. In particular, the concentrations of 5 macronutrients (Ca, K, Mg, P, S), 10 micronutrients (Co, Cr, Cu, Fe, Mn, Na, Ni, Si, V, Zn), and 4 non-essential elements (Al, As, Cd, Pb) were measured. The method precision, calculated as relative standard deviation, based on *n* = 9 sequential measurements of the same sample for each PTE, ranged 2–7%, depending on the element.

For anion analysis, samples were filtered through cellulose filters (Ø 47 mm, 0.2 μm pore size; Whatman, UK) and quantification of ${\mathrm{Br}}^{-}$, ${\mathrm{Cl}}^{-}$, $F^{-}$, ${\mathrm{NO}}_{2}^{-}$, ${\mathrm{NO}}_{3}^{-}$, ${\mathrm{PO}}_{4}^{3-}$, and ${\mathrm{SO}}_{4}^{2-}$ was performed through Ion-Exchange chromatography, using a Dionex IonPac AS22 250 mm × 4 μm column (Thermo, Waltham, MA, USA), with a 50 mm security guard, on a Aquion chromatography system (Thermo, USA). Eluent was constituted by a ${\mathrm{Na}}_{2}{\mathrm{CO}}_{3}$:${\mathrm{NaHCO}}_{3}$ solution ( 4.5 mmol L^−1^:1.4 mmol L^−1^), flushed at 1.20 mL min^−1^ flow rate and pressure ≤1800 kPa.

### 2.4. Sediment Analysis

At each site, sediment sampling was performed across the algae meadow, where 1–2 kg of sediments from the 0–3 cm layer were manually collected using plastic bags, limiting the loss of fine particles. Back in the laboratory, sediments were placed in boxes built of filter paper (in order to facilitate water loss), dried at 105 °C in an incubator (ISCO 9000, Sil.Mar Instruments, Italy) until constant weight, and sieved through 2 mm mesh-sized sieves (AS200 basic, Retsch, Haan, Germany) to retrieve the granulometric fraction. An aliquot of the sieved sediments was pulverized in agate mortars using a planetary ball mill (PM4, Retsch, Germany).

pH and organic matter were determined in triplicate using the granulometric fraction. pH was determined in a water suspension (1:2.5, *w*:*w*, sediment:distilled water) via the potentiometric method (FiveGo, Mettler Toledo, Columbus, OH, USA), whereas organic matter concentration was determined via the gravimetric method, after calcination ( 550 °C for 4 h) in muffle (Controller B 170, Nabertherm, Lilienthal, Germany) of oven-dried samples.

Mineralogical analysis was performed on the pulverized granulometric fraction by means of X-ray diffraction analysis, using a D2 PHASER (Bruker Corporation, Billerica, MA, USA) benchtop XRD system. In particular, samples were placed in plastic holders for powder analysis and X-ray diffractograms were acquired at a step resolution of 0.006° in a 2θ range of 5.002–65.004°, with a PSD opening of 5.002° and a time step of 0.100 s, with continuous scanning. The Cu anode was kept at 30.0 kV, with a current of 10.0 mA. The identification and estimated abundances of the major mineralogical components in the sediment samples were then obtained through Rietveld refinement of the diffractograms, using Profex 5.2.0 [53] software and data from the Crystallography Open Database (http://www.crystallography.net, accessed on 21 March 2022) and the RUff (http://rruff.info/, accessed on 21 March 2022) databases.

The particle distribution size of the granulometric fraction was determined using the sieving method (AS200 basic, Retsch, Germany), by means of sieves with 1000 μm, 500 μm, 250 μm, 125 μm, 63 μm, 38 μm, 20 μm and <20 μm meshes.

### 2.5. Data Analysis

The length and width of internodes, spine cells, corticated and ecorticated branchlet segments, oosporangia, and coronulas were employed as morphological traits characterizing thalli in the data analysis, with the addition of the cortification of the branchlet segments below nodes where gametangia develop (corticated/ecorticated) and of the internodes (haplostichous/diplostichous/triplostichous and isostichous/aulacanthous/tylacanthous). The assignment of each thallus to a species was performed through fuzzy partitioning, using a Gower distance metric for computation of the dissimilarity matrix. The number of clusters was chosen according to the number of candidate taxa derived in the morphological examinations, and an exponential membership coefficient equal to 1.5 was adopted in order to provide an optimal compromise between partitioning crispness and fuzziness. The analyses were performed using the functions of the “cluster” [54] package for the R 4.2.1 programming language [55], visualizing membership probabilities through a ternary diagram drawn with the functions of the “ggtern” [56] package. The role of different morphometric traits in identifying species and differentiating morphotypes within each species was evaluated through recursive partitioning using conditional inference [57], using the functions of the “partykit” [58] package. The relative differentiation among taxa in relation to their traits and to their ecological niches was evaluated through non-metric multidimensional scalings (NMDS) based on 2 axes, with the superimposition of minimum convex polygons for the species. In particular, the distance among populations based on morphological traits, pigment concentrations, epiphyte diatom mass, and carbonate encrustation was calculated using the Gower distance metric, whereas that based on the water and sediment characteristics of the respective growing sites was calculated through the Manhattan distance metric.The analyses were performed using functions of the “vegan” package [59].

## 3. Results

**Figure 2:**
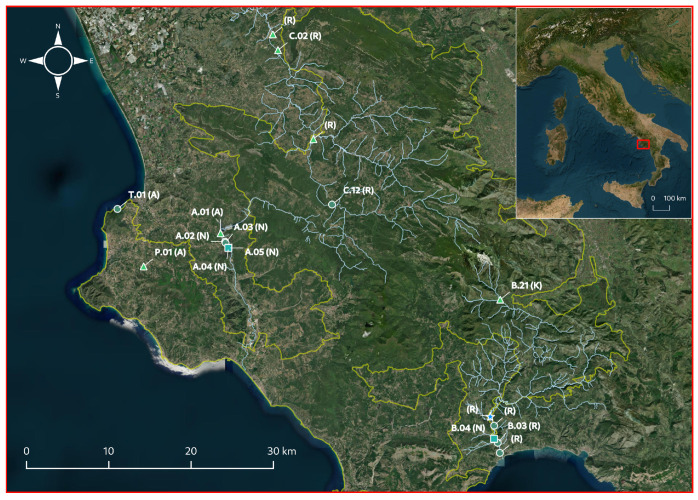
Distribution of the charophyte populations discovered in the study area (red box), with indication of the species: *C. globularis* (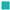), *C. gymnophylla* (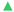), *C. vulgaris* (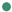) and *C. vulgaris* var *papillata* (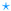), and the type of environment (A: artificial ponds, K: karst resurgences, N: natural ponds, R: rivers). Numbered populations, labeled according to previous studies conducted in the area [39,40,60], are the ones on which the ecological, morphological, and biochemical traits were investigated. Cyan and yellow lines indicate the course of the main rivers in the area and the boundaries of the “Cilento Vallo di Diano and Alburni” National Park, respectively.

Overall, 17 charophyte populations were observed within the freshwater ecosystems of the “Cilento, Vallo di Diano e Alburni” National Park and its neighborhood, all belonging to the *Chara* genus (Figure 2), due to the fully corticated primary axis, stipulodes arranged in 2 rows, and antheridia developing below oogonia [45,46].

The populations were discovered along the course of the main rivers of the area, the Alento, Bussento, and Calore Salernitano, as well as from ponds on the north-western area of the National Park. In particular, 8 populations colonized riverbeds in areas of slowly flowing water, 5 populations colonized lateral ponds along the course of the main rivers, 3 populations colonized artificial ponds with stone bottoms and walls (fountain pools and ancient ponds used for irrigation), and one population colonized a karst resurgence. The populations were invariably observed in oligotrophic waters, as indicated by the concentrations of ${\mathrm{PO}}_{4}^{3-}$ and photosynthetic pigments always below the limits of detection and the concentrations of P in the range 14.2–85.8 μg L^−1^.

In terms of species associations, charophytes were the only macrophytes observed in all the artificial ponds and the karst resurgence, as well as in a lateral pond along the Bussento River and in two sites along the Calore Salernitano River, one in the middle course and one in the upper course. In the other sites, charophytes associated with several rooted hydrophytes and helophytes: *Potamogeton* spp. and *Zannichellia palustris* L. in the lower course of the Calore Salernitano River, *Groenlandia densa* (L.) Fourr. in the lower course of the Bussento River, and *Juncus* spp., *Phragmites australis* (Cav.) Trin. ex Steud. and *Typha angustifolia* L. in the lateral ponds along the Alento River. Whereas, in river environments, charophytes and the associated hydrophytes formed heterospecific mats with a high degree of spatial interspersion among species, in the lateral ponds along the Alento River, charophytes formed pure and dense mats in the central section of the ponds, spatially separated from the vegetation colonizing the banks, among which only a few specimens of *Chara* sp. could be observed. In a single lateral pond along the Alento River, a strict association of *Chara* sp. with *Utricularia gibba* L., a cosmopolitan carnivorous hydrophyte, was observed [61], with deeply intertwined specimens of both the species.

Population traits of 12 of the discovered populations were investigated, due to the disappearance of the others over the years. With one exception of a population in the middle course of the Calore Salernitano River, the disappeared populations colonized sites close to the studied ones (Figure 2) and had morphotypes similar to those of the studied populations. The unique exception was a population in the lower course of the Bussento River (Figure 2) with diagnostic traits typical of *C. vulgaris* (diplostichous, aulacanthous, single spine cells, gametangia between and at the top of corticated branchlet segments), but with spine cells 2–3-times longer than the axis diameter, suggesting identification as *C. vulgaris* var *papillata* K. Wallroth. Remarkably, all the populations that disappeared during the study period colonized the riverbed of the Bussento and the Calore Salernitano Rivers, whereas no population was lost from the ponds, either the natural or the artificial ones (Figure 2).

**Figure 3:**
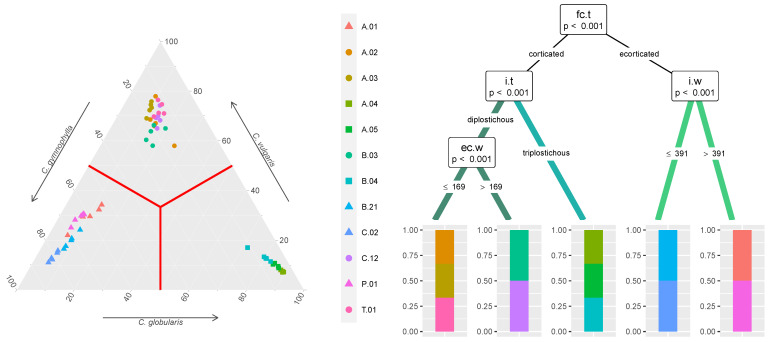
Ternary plot (**left**) with the fuzzy clustering probabilities of each thallus, colored according to their provenances (labeled according to Figure 2), belonging to the three species. Thalli are indicated by different symbols in relation to the most probable species they belong to (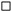: *C. globularis*, 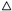: *C. gymnophylla*, 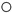: *C. vulgaris*). Populations belonging to each species, indicated by different colors (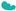: *C. globularis*, 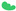: *C. gymnophylla*, 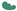: *C. vulgaris*) are further differentiated in the conditional inference tree (**right**) based on their morphological traits (values in μm), labeled according to Table 1 and Figure 1 and indicated on the nodes with their respective *p*-values and the splitting rules.

All the morphotypes observed in the area could be classified into three candidate taxa at the species level: two belonging to section *Chara* R.D. Wood according to the diplostichous cortex, *Chara vulgaris* Linné 1753 and *Chara gymnophylla* A. Braun 1835, and one belonging to section *Grovesia* R.D. Wood according to the triplostichous cortex, *Chara globularis* Thuillier 1799. The average morphological traits per population are reported in Table 2, whereas the assignment of thalli collected from the different populations to the three taxa is reported in Figure 3. Pure populations were invariably observed in each site, although with different degrees of morphological variability.

*C. vulgaris* was the most widely distributed taxon, constituting 5 out of 12 populations, followed by *C. gymnophylla*, with four populations, and *C. globularis*, with three populations. These three species were clearly distinguished by the cortification type of the internodes and by the presence of gametangia between, or on top of, corticated *vs* ecorticated branchlet segments, with additional intra-specific differentiations both in *C. vulgaris* and *C. gymnophylla* related to the morphology of branchlet ecorticated segments and internodes, respectively (Figure 3). In particular, a remarkable differentiation was observed in both *C. vulgaris* and *C. gymnophylla* between populations colonizing rivers (B.03, C.12 and B.21, C.02 respectively) and those colonizing ponds (A.02, A.03, T.01 and A.01, P.01, respectively), irrespective of the geographical location.

**Figure 4:**
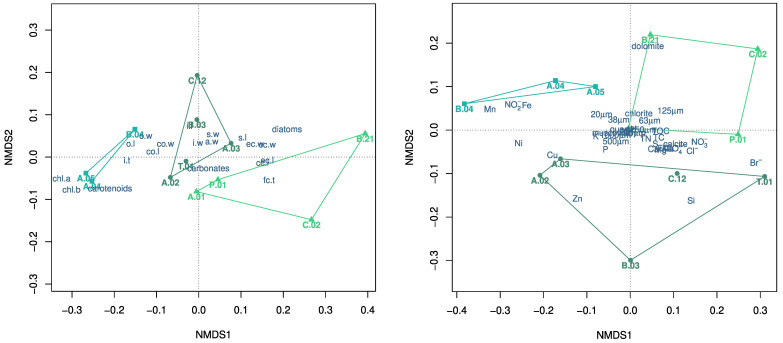
NMDS biplots based on average population traits (**left**) and environmental characteristics (**right**), with the superimposition of the minimum convex polygons grouping the species, indicated by different colors and symbols (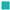: *C. globularis*, 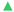: *C. gymnophylla*, 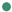: *C. vulgaris*). Abbreviations for populations are reported in Figure 2, with those indicating morphological traits in Table 1 and Figure 1, whereas those indicating environmental characteristics in Section 2.3 and Section 2.4.

The differentiation of morphotypes from rivers and ponds was confirmed using the results of the NMDS analysis, with the former located at higher distances from the origin than the latter in the NMDS space (Figure 4). Moreover, the provenance appeared to differentiate the populations of *C. globularis*, with the population in a lateral pond along the Bussento River (B.04) separating from the others colonizing ponds along the Alento River (A.04, A.05) (Figure 4).

Remarkably, river morphotypes tended to be characterized by higher diatom colonization than the morphotypes from ponds of both *C. vulgaris* and *C. gymnophylla*. The differentiation between river and pond morphotypes did not reflect similar differentiations in the analyzed chemical and physical characteristics of the environment. However, the niches of the three species were clearly separated (Figure 4), notwithstanding the overlapping elevation ranges (5–300 m a.s.l. for *C. vulgaris*, 15–75 m a.s.l. for *C. globularis*, and 70–475 m a.s.l. for *C. gymnophylla*). The main differentiation among the species appeared to be related, in addition to the traits highlighted by recursive partitioning (Figure 3), to the higher pigment concentrations in *C. globularis*. Such an occurrence is associated with the presence of higher concentrations of ${\mathrm{NO}}_{2}^{-}$, Fe and Mn in the water of the environments colonized by this species. Remarkably, the intra-specific variability in species traits and environmental characteristics showed opposing trends between *C. vulgaris* and *C. gymnophylla*, with the former characterized by a reduced plasticity, in spite of more heterogeneous environments being colonized and the opposite being true for the latter (Figure 4). The heterogeneity in the environments colonized by *C. vulgaris* appeared to be primarily associated with the concentrations of Cu, Ni, Si, and Zn in water, whereas the characteristics of sediments, such as the mineralogical composition, the particle size distribution, and the organic matter content, contributed marginally to the variability among sites. An exception in this context was represented by the higher abundance of dolomite in the sediments of one site (B.21) in the higher course of the Bussento River, colonized by *C. gymnophylla*.

## 4. Discussion

The diversity of charophytes in the Mediterranean area under study was relatively low, both in terms of the total number of populations discovered, only 17 over 3 years of field observations, with 12 represented by stable populations, and of the number of taxa they belonged to, amounting to less than one tenth of the species recognized in the Italian flora [45], despite the abundance of wetlands in the area. Such an occurrence contrasts with the high species richness observed [7] in deep volcanic lakes in Italy located within 2° N of the study area, as well as in the two largest Italian islands, Sardinia [1] and Sicily [62]. On the one hand, the higher diversity observed in deep volcanic lakes suggests that the type of colonized environment could be a primary driver of charophyte biodiversity, rather than the geographical location. Such an hypothesis is further supported by the similarly reduced biodiversity observed in Israel where, remarkably, charophytes tend to colonize the same environments [63] as in the area investigated in the present research. On the other hand, the occurrence of several species in temporary freshwater environments in both Sardinia and Sicily [1,62] unobserved in the study area, especially taxa belonging to the Nitelleae tribe, indicates that other drivers, likely eutrophication or land use changes (locally occurring, despite of the general good ecological status of the area [39,40,60]), could be responsible for the reduced diversity of charophytes in the area. Indeed, among the stable populations, all the species observed in the area of the “Cilento, Vallo di Diano e Alburni” National Park, with *C. vulgaris* being the most frequent, followed by *C. gymnophylla* and then by *C. globularis*, invariably occupied oligotrophic rivers and ponds. The low nutrient concentrations were further highlighted by the occasional concurrent presence of a carnivorous plant, *Utricularia gibba*, characteristic of oligotrophic waters [61], suggesting that eutrophication may be a primary driver of species distribution in the area. The disappearance from river sections of almost one third of all populations, however, amounting to two thirds of the populations observed in these environments, indicates that the dynamics in water regime, sedimentation, and shading by riverbank vegetation may be also responsible for their limited persistence. Indeed, although competition with hydrophytes such as *Potamogeton* spp., *Z. palustris* and *G. densa* in the lower course of the Bussento and Calore Salernitano Rivers cannot be ruled out, the disappearance of both angiosperms and charophytes from several sites points toward hydrological dynamics as the primary constraint to the persistence of charophyte populations in rivers. On the one hand, such ephemeral habitats thus appear to be crucial as temporary refugia for Mediterranean charophytes [1]; however, on the other hand, their hydrological dynamics can dramatically shape meta-population dynamics, with potential impacts on long-term conservation of species.

The capability of charophytes to exploit temporary refugia is related to the persistence of oospores in sediments and their rapid growth [64,65], being capable of remaining viable for hundreds of years [66], but little is known about the potential effects of such fast meta-population dynamics on species viability and the adaptability to changing environments. It is likely that the most sensitive species may not survive in rapidly changing environments, and this could explain the low species richness in the area. According to the conservation status of these species in the European Red Lists, [7], *C. vulgaris* is considered from least concern to vulnerable, *C. gymnophylla* as critically endangered, and *C. globularis* from vulnerable to endangered. Incidentally, it should be noted that *C. gymnophylla* appears to be particularly threatened in the Czech Republic, the unique Nation listing it in a red list, but is one of the most widely distributed species in Israel [63], suggesting that warmer climates may actually favor this species.

In terms of changing environments, it is possible that the rapid alterations of the quality of freshwater ecosystems, driven by the intensification of agriculture in the latter half of the 20th century, together with the habitat loss due to other anthropogenic activities [3], have contributed to the disappearance of the most fragile species in the study area. Unfortunately, the absence of historical records [45] prevents the evaluation of long-term charophyte dynamics in this area and of the occurrence of local extinction events. Historical reconstructions of past charophyte communities based on sediment oospores or gyrogonites [67] could possibly overcome this issue and warrant further investigations, although the movements of the river paths and sediments represent non-negligible sources of uncertainty. The habitat loss hypothesis, however, is supported by the contraction in species richness in a similar environmental setting, in Israel [63], and by the disappearance of several taxa from historical locations in different Mediterranean countries [27,45]. Anthropogenic activities, however, do not necessarily harm charophyte populations, but can even determine the creation of refugia for particular species, as demonstrated by the colonization of diverse artificial ponds by *C. gymnophylla* and *C. vulgaris*. Such a result corroborates the findings of Panzeca et al. [23] on the colonization of artificial farm ponds in Sicily by *C. globularis* and *C. vulgaris*, and of Becker [1] on the colonization of artificial troughs and reservoirs in Sardinia by the same species, indicating that especially *C. vulgaris*, but also a few other species, actually benefit from the creation of artificial freshwater environments, where it forms stable populations. Remarkably, artificial ponds are among the few environments in the study area where charophyte actually dominated submerged vegetation, with traits typical of the *Charetea intermediae* phytosociological class, and, therefore, of the habitat 3140—Hard oligo-mesotrophic waters with benthic vegetation of *Chara* spp.

The environments colonized not only affected population dynamics and persistence, but also the vegetative structures [14] of the two species occurring in both rivers and ponds, where *C. gymnophylla* and *C. vulgaris* occurred with different morphotypes. The size of gametangia, especially oosporangia, does not appear to vary in relation to the colonized environment, although variations in oospores have recently been reported by Milovanović et al. [38] for *C. globularis* populations growing in different sites in continental Europe, characterized by different water chemistries and stabilities. In this context, the intra-specific differentiation of both *C. gymnophylla* and *C. vulgaris* into different morphotypes, as well as the clear-cut separation between the species is remarkable, considering the long-standing debate on the validity of the former as a taxonomical entity distinguished from the latter [16,45]. At least in the Mediterranean study area, our results indicate that the taxa are actually characterized by a different morphology and ecology, as asserted by other researches [14]. Remarkably, the morphological differentiation among species reflects different interactions with both the abiotic and biotic environment, as highlighted by the variations in photosynthetic pigment concentrations, with *C. globularis* having the highest values, and by the different colonization by epiphytes, with *C. gymnophylla* hosting a higher diatom biomass. Interestingly, among the studied populations, these characteristics appear to be affected by the species more than by the environment, a finding that warrants further investigation to evaluate its local nature or, conversely, its general validity.

From a conservation viewpoint, in spite of the urgency of clarifying charophyte responses to environmental gradients and developing tailored conservation strategies [31], the elucidation of how the environment drives species distribution remains challenging, even when a large number of environmental descriptors are employed, as in the present research. Here, the adopted approach revealed the occupation of different ecological niches by the three species, a conclusion supported by the invariable occurrence of pure populations in the studied ecosystems and that sheds light on the environmental gradients likely shaping their occurrence. In this context, the three species are characterized by different ecological plasticities, which do not mirror the relative morphological plasticities. In particular, *C. vulgaris* is able to colonize more heterogeneous environment, with little variation of its traits, whereas the opposite is true for *C. gymnophylla*. Among the three species, *C. globularis* appears to be the least generalist in the studied area, only being able to colonize ponds with limited heterogeneity in their environmental characteristics.

Overall, geographical characteristics, such as elevation (ranging from 5 to 475 m a.s.l.), represent ecological factors marginally affecting species distribution in the study area, as also reported by Auderset Joye et al. [9] in relation to the occurrence and distribution of Characeae in continental Europe (along an elevation range of 190–2500 m a.s.l.). Indeed, despite several species showing a preference for certain elevation ranges, with the highest community biodiversity at the lowest end, they are likely more sensitive to other environmental factors that vary with elevation, such as temperature, dissolved oxygen, and dissolved organic matter, than to the elevation itself [9]. The variations in environmental factors with elevation would therefore explain the importance of this repeatedly documented variable [65], with a clear example provided by *C. vulgaris* in south Kazakhstan, preferring low altitude habitats with higher water temperatures and organic compounds, as well as lower oxygen and pH [6].

Among the other abiotic factors investigated, which are usually considered more important than the biotic ones for determining charophyte occurrence and distribution [31], water chemical composition and sediment mineralogical composition appeared to shape the occurrence of charophytes in the study area. In particular, the relatively higher concentrations of Fe and Mn in the environments colonized by *C. globularis* was consistent with the colonization of ponds, where the sub-oxic and acidic conditions in sediments tended to favor reduced (and more soluble) forms of these elements [68]. Such an occurrence is supported by the relatively higher concentrations of ${\mathrm{NO}}_{2}^{-}$ instead of ${\mathrm{NO}}_{3}^{-}$. In this respect, the renowned tolerance of *C. globularis* for eutrophic conditions [9], a trait in common with *C. vulgaris* that indicates the opportunistic strategy of both species [37] (the latter is explicitly defined as an R-strategist by Blindow et al. [13]), does not appear to be relevant in the study area, where both the species are invariably restricted to oligotrophic waters. The capability to colonize environments with relatively higher water concentrations of Cu, Ni, Si, and Zn in the study area, however, supports the wide ecological plasticity of *C. vulgaris*, whereas *C. gymnophylla* appears to be more specialized toward calcareous substrates, as reported by Becker [1].

Other reportedly crucial factors in controlling charophyte distribution in coastal ecosystems [31] and of relevance in freshwater ecosystems, such as salinity (as ${\mathrm{Cl}}^{-}$ concentration), seemed to negligibly affect the species distribution in the freshwater ecosystems of the study area. Similarly to our findings, Khuram et al. [28] also reported a marginal effect of salinity (and EC) on the spatial distribution of charophytes in the Peshawar Valley (Pakistan), as well as the tolerance of *C. vulgaris* and *C. globularis* of water pollution.

Overall, such findings are in line and support the evaluations of the conservation status of the three species in Europe, where the distribution ranges of *C. globularis* and *C. vulgaris* are reported to have expanded over the centuries due to their better resistance to anthropogenic stresses [9] and where the conservation of *C. gymnophylla* appears to be more challenging [7].

## Figures and Tables

**Figure 1:**
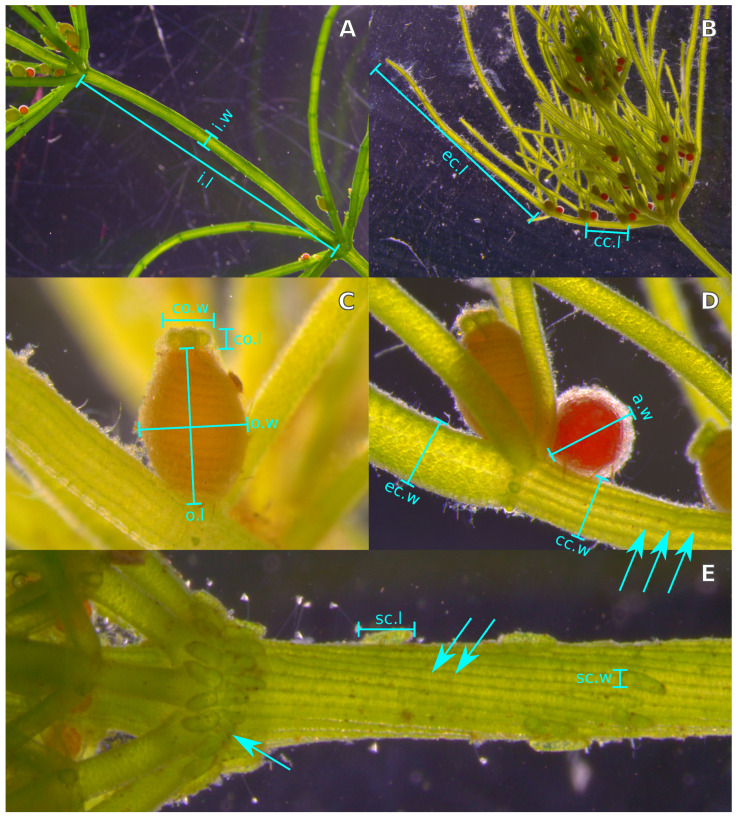
Illustration of the traits reported in Table 1 from exemplary specimens of *C. globularis* (**A**), *C. vulgaris* (**B**–**D**) and *C. gymnophylla* (**E**). Labeled segments indicate how the measurements of length (*.l) and diameter (*.w) were carried out on internodes (**A**), branchlet segments (**B**,**D**), oosporangia and coronula (**C**), antheridia (**D**), and spine cells (**E**). Arrows indicate the type of stipulodes (single arrow), the type of internode cortification (double arrow), and the cortification of branchlet segments below nodes bearing gametangia (triple arrow).

**Table 1:** Morphological traits analyzed for the charophyte populations, with indications of the abbreviations used in the text. The definition of each trait is provided in Figure 1.

	Length	Diameter	Cortification
Internodes	i.l	i.w	i.t ^a^
Arrangement of cortical cells			c.t ^b^
Spine cells	s.l	s.w	
Ecorticated branchlet segments	ec.l	ec.w	
Corticated branchlet segments	cc.l	cc.w	
Oosporangia	o.l	o.w	
Antheridia		a.w ^c^	
Coronula	c.l	c.w	
Branchlet cells below nodes with gametangia			fc.t ^d^
Stipulodes			s.t

^a^ haplostichous/diplosticous/triplostichous, ^b^ isostichous/aulacanthous/tylacanthous, ^c^ due to the spherical shape of antheridia, length and diameter are approximately equal, ^d^ corticated/ecorticated.

**Table 2:** Morphological traits, expressed as mean ± standard deviation in the case of numerical variables, of the thalli from each charophyte population. The types of internode cortification (dip.: diplostichous, tri.: triplostichous; iso.: isostichous, aul.: aulacanthous) and of the cortification of branchlet segments under nodes with gametangia (cor.: corticated, eco.: ecorticated) are also reported. Abbreviations are defined in Table 1 and Figure 1.

	i.l (mm)	i.w (μm)	s.l (μm)	s.w (μm)	cc.l (mm)	cc.w (μm)	ec.l (mm)	ec.w (μm)
A.01	25.3 ± 1.8	500 ± 72	55 ± 14	23.2 ± 7.8	1.23 ± 0.46	176 ± 41	0.99 ± 0.40	151 ± 33
A.02	18.7 ± 1.2	490 ± 100	63 ± 17	37 ± 16	1.26 ± 0.51	166 ± 56	1.13 ± 0.55	154 ± 47
A.03	46.9 ± 2.8	640 ± 160	100 ± 18	44.9 ± 5.7	1.42 ± 0.46	185 ± 42	1.02 ± 0.44	155 ± 48
A.04	12.35 ± 0.66	388 ± 57	49 ± 10	49 ± 11	1.15 ± 0.42	158 ± 27	0.99 ± 0.64	141 ± 47
A.05	24.8 ± 1.7	386 ± 65	73 ± 15	67 ± 43	1.07 ± 0.57	146 ± 28	1.02 ± 0.42	142 ± 32
B.03	23.7 ± 1.7	430 ± 140	87 ± 30	33.3 ± 4.7	1.17 ± 0.66	237 ± 68	1.31 ± 0.72	217 ± 69
B.04	14.3 ± 1.2	292 ± 66	63 ± 23	53 ± 22	1.40 ± 0.51	189 ± 44	1.33 ± 0.61	177 ± 48
B.21	9.67 ± 0.46	315 ± 37	101 ± 40	67 ± 17	2.07 ± 0.73	367 ± 91	2.07 ± 0.74	305 ± 72
C.02	14.3 ± 1.2	360 ± 110	79 ± 36	65 ± 25	2.22 ± 0.69	236 ± 48	2.00 ± 0.94	192 ± 57
C.12	47.4 ± 3.5	750 ± 260	103 ± 23	91 ± 33	1.25 ± 0.51	213 ± 60	1.07 ± 0.59	182 ± 64
P.01	29.8 ± 3.4	560 ± 140	86 ± 31	30 ± 12	1.22 ± 0.49	171 ± 45	1.14 ± 0.37	162 ± 66
T.01	28.4 ± 2.2	466 ± 79	50 ± 12	38 ± 19	1.22 ± 0.43	169 ± 50	1.03 ± 0.64	157 ± 36
	**o.l (** μ **m)**	**o.w (** μ **m)**	**co.l (** μ **m)**	**co.w (** μ **m)**	**a.w (** μ **m)**	**i.t**	**c.t**	**fc.t**
A.01	401 ± 53	297 ± 26	127 ± 19	136 ± 22	250 ± 72	dip.	aul.	eco.
A.02	428 ± 96	227 ± 70	106 ± 37	119 ± 25	184 ± 36	dip.	aul.	cor.
A.03	381 ± 55	210 ± 35	71 ± 17	103 ± 21	207 ± 48	dip.	aul.	cor.
A.04	534 ± 47	314 ± 35	135 ± 30	128.0 ± 8.7	204 ± 21	tri.	iso.	cor.
A.05	580 ± 57	333 ± 43	113 ± 13	122 ± 25	144 ± 64	tri.	iso.	cor.
B.03	514 ± 48	343 ± 25	126 ± 47	165 ± 34	196 ± 13	dip.	aul.	cor.
B.04	566 ± 72	305 ± 49	148 ± 46	130 ± 25	219 ± 34	tri.	iso.	cor.
B.21	276 ± 82	203 ± 70	90 ± 40	98 ± 42	234 ± 67	dip.	iso.	eco.
C.02	166 ± 45	110 ± 38	56.8 ± 8.9	68 ± 13	187 ± 42	dip.	aul.	eco.
C.12	464 ± 37	355 ± 37	97.9 ± 9.6	131 ± 24	306 ± 39	dip.	aul.	cor.
P.01	430 ± 47	226 ± 48	116 ± 20	118 ± 14	194 ± 20	dip.	aul.	eco.
T.01	418 ± 76	248 ± 65	112 ± 19	125 ± 15	228 ± 30	dip.	iso.	cor.

## Data Availability

The data presented in this study and specimens from the studied populations are available on request from the authors.
